# Machine Learning Prediction of Crossbred Pig Feed Efficiency and Growth Rate From Single Nucleotide Polymorphisms

**DOI:** 10.3389/fgene.2020.567818

**Published:** 2020-12-18

**Authors:** Llibertat Tusell, Rob Bergsma, Hélène Gilbert, Daniel Gianola, Miriam Piles

**Affiliations:** ^1^ GenPhySE, Université de Toulouse, National Research Institute for Agriculture, Food and the Environment (INRAE), Castanet-Tolosan, France; ^2^ Topigs Norsvin Research Center, Beuningen, Netherlands; ^3^ Department of Animal Sciences, University of Wisconsin-Madison, Madison, WL, United States; ^4^ Department of Dairy Science, University of Wisconsin-Madison, Madison, WI, United States; ^5^ Animal Breeding and Genetics Program, Institute of Agriculture and Food Research and Technology (IRTA), Barcelona, Spain

**Keywords:** pigs, crossbred, single nucleotide polymorphism, genomic prediction, support vector machine, machine learning

## Abstract

This research assessed the ability of a Support Vector Machine (SVM) regression model to predict pig crossbred (CB) performance from various sources of phenotypic and genotypic information for improving crossbreeding performance at reduced genotyping cost. Data consisted of average daily gain (ADG) and residual feed intake (RFI) records and genotypes of 5,708 purebred (PB) boars and 5,007 CB pigs. Prediction models were fitted using individual PB genotypes and phenotypes (**trn.1**); genotypes of PB sires and average of CB records per PB sire (**trn.2**); and individual CB genotypes and phenotypes (**trn.3**). The average of CB offspring records was the trait to be predicted from PB sire’s genotype using cross-validation. Single nucleotide polymorphisms (SNPs) were ranked based on the Spearman Rank correlation with the trait. Subsets with an increasing number (from 50 to 2,000) of the most informative SNPs were used as predictor variables in SVM. Prediction performance was the median of the Spearman correlation (SC, interquartile range in brackets) between observed and predicted phenotypes in the testing set. The best predictive performances were obtained when sire phenotypic information was included in trn.1 (0.22 [0.03] for RFI with SVM and 250 SNPs, and 0.12 [0.05] for ADG with SVM and 500–1,000 SNPs) or when trn.3 was used (0.29 [0.16] with Genomic best linear unbiased prediction (GBLUP) for RFI, and 0.15 [0.09] for ADG with just 50 SNPs). Animals from the last two generations were assigned to the testing set and remaining animals to the training set. Individual’s PB own phenotype and genotype improved the prediction ability of CB offspring of young animals for ADG but not for RFI. The highest SC was 0.34 [0.21] and 0.36 [0.22] for RFI and ADG, respectively, with SVM and 50 SNPs. Predictive performance using CB data for training leads to a SC of 0.34 [0.19] with GBLUP and 0.28 [0.18] with SVM and 250 SNPs for RFI and 0.34 [0.15] with SVM and 500 SNPs for ADG. Results suggest that PB candidates could be evaluated for CB performance with SVM and low-density SNP chip panels after collecting their own RFI or ADG performances or even earlier, after being genotyped using a reference population of CB animals.

## Introduction

Feed efficiency and growth rate are two of the most important components of productivity and sustainability of meat production. Many meat production livestock systems rely on crossbred (CB) animals (pig, poultry, rabbits, and some beef cattle systems), but the genetic improvement of these traits commonly takes place in purebred (PB) lines based on PB performance only. However, the ultimate goal of selection is achieving competitive performances in CB animals raised in commercial farms. The genetic gain attained from within line selection in the PB line will not be efficiently transferred to the CB population if the genetic correlation between PB and CB performances ($r_{\mathrm{PB},\mathrm{CB}}$)differs markedly from unity. A low correlation might be due to genotype by environment interactions or presence of non-additive genetic effects (Wei and van der Steen, 1991). For feed efficiency (FE) and growth traits in pigs, the average estimate of $r_{\mathrm{PB},\mathrm{CB}}$ is 0.66 across 27 studies reviewed (Wientjes and Calus, 2017). This moderate $r_{\mathrm{PB},\mathrm{CB}}$ value indicates that accounting for CB information in genetic evaluation of pig PB lines would be a reasonable strategy to boost CB performance (Wei and van der Werf, 1995).

With the availability of high-density single nucleotide polymorphism (SNP) genotype data, several parametric genomic selection (GS) models can be used to evaluate candidates for improved PB and CB performances. Some of the proposed parametric models account for additive genetic effects only (Ibañez-Escriche et al., 2009; Christensen et al., 2014, 2015; Tusell et al., 2016). Other models include both additive and dominance effects using either genomic information from PB animals (Esfandyari et al., 2016) or treating PB and CB data as different traits (Vitezica et al., 2016; Xiang et al., 2016). These models differ in complexity and type of phenotypic and genotypic information required. To our knowledge, non-parametric GS models that account for non-additive genetic effects have not been proposed yet in the PB-CB context. Finding a suitable genome-enabled prediction model fitted at a reduced genotyping cost, but still capable of predicting yet-to-be observed two‐ or three-way CB FE and growth performances from PB genotypes, is of great interest.

Machine learning methods could be useful for CB performance prediction purposes because of their ability to predict outputs without assumptions about the genetic determinism underlying a trait. This property can be relevant for predicting CB performance because of the need to accommodate non-additive genetic effects. Machine learning methods are increasingly used when the number of parameters is much larger than the number of observations, as it is the case of high-throughput datasets such as those with high-density genetic markers for GS. Machine-learning models that are non-linear in either predictor variables or parameters have been proposed in animal and plant breeding to enhance genome-enabled prediction of complex traits (Gianola et al., 2006, 2011; Gianola and van Kaam, 2008). Among them, a support vector machine is regarded as one of the most efficient machine learning algorithms, and it has been used successfully in many different fields (James et al., 2013; Attewell et al., 2015) including livestock and plant breeding (Moser et al., 2009; Long et al., 2011; Montesinos-López et al., 2019).

Feature selection, i.e., selection of a subset of predictor variables from the input data, reduces computation requirements and negative effects on prediction performance of irrelevant variables *via* over-fitting, an especially important matter in studies with high-dimensional/high-throughput data (Chandrashekar and Sahin, 2014). Finding a prediction model able to perform well with a small subset of SNPs can be of interest to predict CB performance from low-density SNP chips. In particular, the possibility to evaluate selection candidates of the PB lines for improved CB performance at a low genotyping cost, especially if a CB reference population is needed, is of great interest.

The goal of this research was to assess the ability of support vector machine (SVM) regression model trained with different sources of phenotypic and genotypic information to predict CB feed efficiency and growth rate in pigs. The ultimate objective is to design potential strategies for improving pig crossbreeding productive performance at reduced genotyping cost.

## Materials and Methods

All data used in this study were obtained from existing database made available by Topigs Norsvin (Beuningen, Netherlands). Therefore, no Animal Care Committee approval was necessary for the purposes of this study.

### Animals

Animals were produced by Topigs Norsvin (Beuningen, Netherlands). They consisted in 5,708 boars from a terminal sire line (PB) and 5,007 three-way CB growing-finishing pigs (CB, 3,399 males and 1,608 females) originated from the cross of 348 PB boars and 621 sows from two different maternal lines to produce the commercial CB sow, sired by the PB terminal sire line. All PB animals were born and raised in two specific pathogen free nucleus farms, one of them located in the Netherlands, the other one in France. All CB animals were born and raised in two commercial farrows to finish farms in Netherlands. Semen exchange between both nucleus farms takes place routinely. Semen of the (PB) terminal sire line used to produce the CB pigs predominantly originated from sires born on one of the two nucleus farms.

Both nucleus farms as well as both farrow to finish farms were equipped with IVOG feeding stations (INSENTEC, Marknesse, Netherlands) that register individual feed intake of group housed pigs. All pigs had ear tags with unique numbering; therefore, individual feed intake records were available for all pigs for each day on test. The pigs were fed with *ad libitum*, a commercially available diet, until the end of the performance test (PB) or throughout their entire life (CB).

### Phenotypes

Average daily gain (ADG, g/day) was measured for PB animals between the beginning (median age of 68 days and median weight of 31 kg) and the end of the test (median age of 155 days and median weight of 130 kg). ADG was measured for CB animals between the start of the grower-finisher period (median age of 68 days and median weight of 25 kg) until the day before slaughter (median age of 173 days and median weight of 124 kg). Only records from PB/CB animals starting the test/grower-finisher period between 50 and 105 days of age and remaining on test/grower-finisher period between 60 and 120 days were retained.

Backfat thickness was determined ultrasonically on live animals (US-fat in mm) in PB animals at the end of the test period and on carcass with the Capteur Gras Maigre device (Sydel, in mm) in CB animals. Metabolic weight (g) was calculated as $MW={\left(\left(W_{start}+W_{end}\right)/2\;\right)}^{0.75}$, where $W_{start}$ and $W_{end}$ are the weights at the beginning and at the end of the test period, respectively.

Among all PB and CB data available, three subsets of data were considered: (i) individual phenotypes from genotyped PB individuals (dPB), (ii) individual CB phenotypes that were offspring of genotyped PB sires (dCB_SIRE_), and (iii) individual phenotypes from genotyped CB individuals (dCB). Notice that some PB sires originating dCB_SIRE_ records had their own dPB records and that dCB included only genotyped CB animals.

Separately in each data subset, multivariate outlier records of ADG, daily feed intake, backfat thickness, and metabolic weight were identified and removed within batch, farm and sex (only for CB records) when the squared Mahalanobis distance to the center of the distribution was >12 (Drumond et al., 2019). Then, residual feed intake (RFI) was estimated as the residual of a linear regression of daily feed intake on average daily gain, backfat thickness, and metabolic weight (lm function, R Core Team, 2019). After that, phenotypes of ADG and RFI were pre-adjusted by environmental effects, fitting a linear model (lm function, R Core Team, 2019) for each data subset. The model included the effects of age at the start of the test (covariate), duration of the performance test (covariate), and the combination of farm and batch (**farm × batch**) and sex (only included in the CB data subsets). The farm × batch effect resulted from the combination of two farms and 2 month period batches for both PB and CB data. Only farm × batch levels with ≥10 records were retained for the analyses. The adjusted records for the three data subsets were obtained after subtracting the estimates of these systematic environmental effects to the original traits. The average of adjusted CB records per PB sire was calculated in the dCB_SIRE_ dataset (median of number of offspring records per sire was of 10 with a SD = 11.8).

Table 1 shows the number of records available for each dataset and summary statistics of the phenotypes.

**Table 1:** Mean (SD in parentheses and range in square brackets) of residual feed intake (RFI) and daily gain (DG) at fattening for the three data subsets.

Data subset description (abbreviation)	RFI (g/day)	Average daily gain (ADG; g/d)	Number of records	Farm × batch levels	Males/Females	Sires/dams
Individual phenotypes from genotyped PB individuals (dPB)	−44 (216) [−710, 635]	1112 (128) [688, 1,508]	5,708	46	5,708/0	217/1120
Individual phenotypes of CB offspring from genotyped PB sires (dCB_SIRE_)	71 (169) [−437, 611]	877 (88.55) [566, 1,265]	3,495 from 257 sires	47	2,520/975	257/490
Individual phenotypes from genotyped CB individuals (dCB)	0.10 (150.10) [−518, 583]	885.5 (87.39) [541.0, 1285.0]	3,197	53	2262/935	252/478

### Genotypes

Animals were genotyped using the Illumina Porcine SNP60 BeadChip (Illumina, Inc., San Diego). SNPs with a call rate lower than 0.90 and a minor allele frequency lower than 0.05 were removed from the whole genotype dataset. Animals with a call rate lower than 0.90 and parent-offspring pairs that displayed Mendelian inconsistencies were discarded. After this quality control, 46,610 SNPs were retained to pursue the analyses. Separately in each data subset, zero and near-zero-variance predictors were identified and removed with the nearZeroVar function with a cut-off for the ratio of frequencies for the most common value over the second most common value of 95/5 (Caret R package, Kuhn, 2008). Subsequently, the findCorrelation function (Caret R package, Kuhn, 2008) with a cut-off = 0.8 was used to diminish highly pair-wise correlations between features. After this genotype edition, 9,523 SNPs were retained for the PB individuals from the dPB dataset and 9,533 SNPs for the PB sires from the dCB_SIRE_ dataset. Genotypes from the CB individuals of the dCB dataset were trimmed keeping the same 9,533 SNPs retained for the PB sires to ensure, for predictive purposes, that the SNPs were also segregating in the PB line.

### Information Used for Model Fitting and Prediction

**Figure 1:**
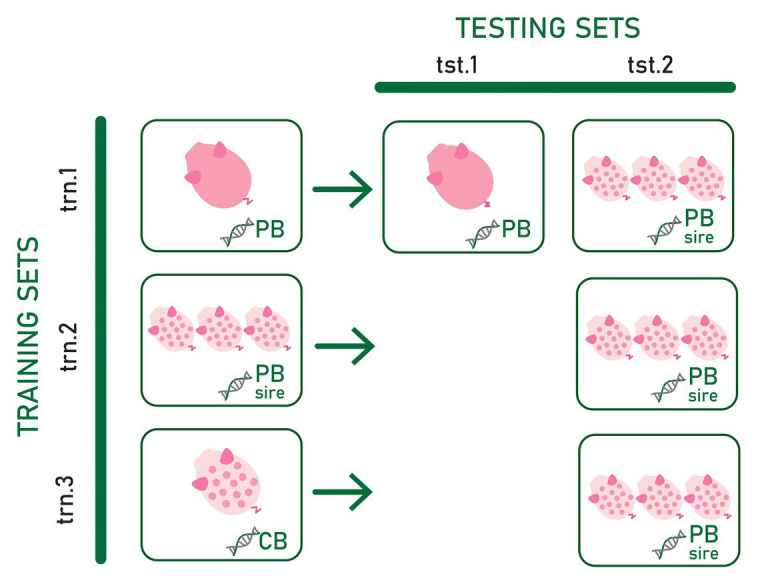
Three types of training sets (trn.1, trn.2, and trn.3) and two types testing sets (tst.1 and tst.2) differing in the phenotype and genotype data used to train and test the prediction models. Pink pig and spotted white pig represent individual purebred (PB) and crossbred (CB) phenotype records, respectively. Three spotted white pigs represent average CB offspring phenotype records per PB sire. DNA chains with “PB,” “CB,” and “PB sire” represent the genotype of the PB animal, the genotype of CB animal, and the genotype of the PB sire of the CB offspring, respectively.

Three types of training sets and two types of testing sets differing in the type of genotype and phenotype information included were used to assess the most convenient phenotypic and genotypic data to predict CB pig feed efficiency and growth rate for establishing a suitable strategy to select PB candidates for improved CB performance. The evaluated scenarios are summarized in Figure 1.

In the first training set (**trn.1**), genotypes from PB animals were used as predictor variables of their own adjusted RFI or ADG record (dPB). In the second training set (**trn.2**), genotypes of the PB sires were the predictor variables for the target response of average of adjusted CB records per PB sire (dCB_SIRE_). Thus, in this training set, average CB offspring performance was considered a PB sire’s trait. Finally, the third training set (**trn.3**) consisted of individual CB adjusted phenotypes and genotypes (dCB). Thus, in trn.1, the reference population in which the model is fitted is exclusively composed of information from the PB animals. In trn.2, the model is fitted on phenotype records of CB pigs using genotype information from PB animals. Finally, in trn.3, the model is fitted exclusively using individual CB information.

The first testing dataset (**tst.1**) consisted of yet-to-be observed PB adjusted records (target trait) that were predicted from the own individual PB genotype (dPB). The second testing set (**tst.2**) consisted of yet-to-be observed average of adjusted CB offspring records per PB sire (target trait) to be predicted from the sire genotype.

The combination of trn.1. and tst.1 (scenario **trn.1-tst.1**) allows to know the within PB line prediction quality when own individual PB genotypes and phenotypes are used. This is considered as the benchmark result because current selection strategies are based on PB individual prediction. The tst.2 in combination with trn.1, trn.2, and trn.3 allowed assessing the most convenient phenotypic and genotypic data to predict CB pig feed efficiency and growth rate. The combination of trn.1 and tst.2 (scenario **trn.1-tst.2**) allows assessing the ability of the PB sire genotype to predict their average CB offspring performance when the prediction model is fitted using individual PB phenotypes and genotypes. In this case, the own phenotype and genotype information of the sires from whose CB offspring performance are predicted may be present or not in the training data, which could have consequences on the quality of prediction. The PB candidates could be evaluated either right after being phenotyped themselves or even before (when only their genotypes are available). If predictions are accurate enough, the resultant fitted model could be used to improve CB performance by selection in PB lines very early in time without the need of CB progeny and CB genotypes. The fitted model obtained in trn.2 requires progeny records available from PB sires. Combined with tst.2 (scenario **trn.2-tst.2**), it could be used to improve CB performance by selection of PB lines in the case that genes involved in growth rate and feed efficiency differ between PB and CB populations. Finally, the scenario resulting from the combination of trn.3 with tst.2 (**trn.3-tst2**) explored the feasibility of using a CB reference population to fit a model to be used for predicting CB progeny performance from PB sire genotypes. This strategy would allow selecting PB lines for improved CB performance when CB and PB performances have a different genetic determinism (e.g., presence of relevant non-additive variance and, therefore, potential heterosis, Esfandyari et al., 2015) while evaluating PB candidates early in time. However, it requires genotyped and phenotyped CB animals, which is not a common practice in pig breeding schemes.

### Model Fitting and Assessment of Predictive Performance

For all scenarios and different combinations of prediction method (i.e., learner) and SNP subset size, model fitting and hyper-parameter optimization were conducted with a nested cross-validation. Nested cross-validation allows estimating the generalization error of the underlying model and its hyper-parameter search (Bischl et al., 2016). It consists of several training-validation and testing dataset splits. An outer k-fold cross-validation using all data was performed using k-1 equal size parts of the original data sets for training the model, and the remaining one for testing. Hyper-parameter tuning was performed in an inner cross-validation within each outer training fold. Same data split (i.e., same data subsets) was used across combinations of learners and datasets to compare prediction performance in the same conditions regarding data structure and composition.

Within each outer training set, features (i.e., SNPs) were standardized and selected according to a ranking based on the Spearman Rank correlation between the feature and the target trait. Different subsets with increasing number (50, 250, 500, 750, 1,500, and 2,000) of the most correlated SNPs were selected. For each of those SNP’s subsets, a SVM regression model (explained in more in detail in the “Learner” section below) was fitted to the corresponding training set after identifying the optimal hyper-parameters in an inner 6-fold cross-validation.

Model fitting and assessment of predictive performance in trn.1-tst.1 scenario was conducted with an outer 10-fold cross validation randomly splitting dPB into 10 folds. Within each of these 10 folds, standardization of the predictor variables using the mean and SD from the corresponding training set was first carried on in both the training and testing sets. Then, the prediction performance of the model fitted with trn.1 was also evaluated in tst.2 separately for (i) the CB sires in tst.2 whose own individual performance also appeared in the trn.1 training set (“**IN training sires**”) and (ii) from those CB sires in tst.2 that did not intervene in the trn.1 training set (“**OUT of training sires**”). Model fitting and assessment of predictive performance in trn.2-tst.2 and trn.3-tst.2 combinations were conducted with an outer 5-fold cross validation repeated five times because of the smaller amount of available data. In trn.2-tst.2 scenario, the average of adjusted CB records of the PB genotyped sires (257 records from dCB_SIRE_) was randomly split into five approximately equal subsets. In scenario trn.3-tst.2, the 5-fold was obtained, ensuring that sires with records in the testing set had no individual CB progeny records in the training set of the same fold. Feature standardization in all of those testing sets was carried on using their own information (i.e., the mean and standard deviation of the SNPs).

The predictive performance of the models in the testing sets was evaluated in terms of accuracy, as the Spearman correlation between the true and the predicted trait across the *k* outer testing sets (SC), and in terms of stability/generalizability of the results, as the interquartile range (IQR) of those values.

### Prediction Performance in the Youngest Generations

Predictive performances obtained in trn.1-tst.1, trn.1-tst.2, trn.2-tst.2, and trn.3-tst.2 using *k*-fold cross-validation allowed evaluating not only the predictive ability but also the stability of results (i.e., sensitivity to changes in the data set) from models fitted using different types of phenotype and genomic information. In a breeding program, the aim is to predict the productive performance of the selection candidates belonging to current generation from data coming from individuals of previous generations. Trying to emulate this situation, for each scenario animals from the last two generations (**YOUNG**) were assigned to the testing set, whereas the remaining ones (**OLD**) were used in the training set. Animals were assigned to a generation using the pedigree R package (Coster, 2013) using their pedigree information. Table 2 shows the amount of records and the number of generations available in the training and testing sets. Notice that because of data were split by generation, only a single prediction per scenario was obtained (i.e., no cross-validation was performed). Thus, for each SNP subset, models were fitted in a unique training dataset, after hyper-parameter tuning by 6-fold cross-validation, and tested on a unique testing set corresponding to the two latest generations. Accuracy of prediction was measured as the Spearman correlation between observed and predicted phenotype, with its median and IQR assessed through a bootstrap approach (Efron, 1981). Pairs of predicted and observed phenotypes in the testing set were assumed to be independent and identically distributed. Pairs corresponding to the number of individuals in the testing set were sampled with replacement from the whole testing set 500 times, and the Spearman correlation was computed in each of the 500 bootstrap samples. Denote these new scenarios as **trn.1**_**OLD**_**-tst.1**_**YOUNG**_, **trn.1**_**OLD**_**-tst.2**_**YOUNG**_, **trn.2**_**OLD**_**-tst.2**_**YOUNG**_, and **trn.3**_**OLD**_**-tst.2**_**YOUNG**_. Dataset **trn.1**_**OLD**_ contained individual phenotype and genotype information of PB OLD animals. Dataset **trn.2**_**OLD**_ included average adjusted CB offspring records from PB OLD sires. Dataset **trn.3**_**OLD**_ consisted of individual phenotype and genotype information of CB OLD animals. Dataset **tst.1**_**YOUNG**_ contained individual phenotype and genotype information of PB YOUNG individuals and dataset **tst.2.**_**YOUNG**_ included average adjusted CB YOUNG offspring records from PB sires. Then, the prediction performance of the model fitted with trn.1_OLD_ was evaluated in **tst.2.**_**YOUNG**_ separately for (i) the CB sires in **tst.2.**_**YOUNG**_ whose own individual performance also appeared in the trn.1_OLD_ training set (“**IN training sires**”) and (ii) from those CB sires in **tst.2.**_**YOUNG**_ that did not intervene in the trn.1_OLD_ training set (“**OUT of training sires**”).

**Table 2:** Number of records and generations included in the different types of training (trn) and testing sets (tst).

	trn.1_OLD_	trn.2_OLD_	trn.3_OLD_	tst.1_YOUNG_	tst.2_YOUNG_
Number of records	3,209	3,059 from 206 PB sires	3,998	2,499	436 from 51 PB sires
Number of generations	5	5	9	2	2

### Learner

SVM for regression was used as learner. It aims at identifying, for a set of prediction variables (*x*), a function that has a maximum deviation *ε* from the observed values (*y*) and has a maximum margin. SVM generates a model representing a tube with radius *ε* fitted to the data. A complete review on this method can be found in Smola and Schölkopf (2004). The power of the SVM resides in a particular mathematical element known as kernel. One of the most used kernel is the Gaussian Radial Basis (RBF) because almost every surface can be obtained with it (Christianini and Shawe-Taylor, 2000). One of the main parameters in a SVM is the “cost parameter” (C), which is a trade-off between the prediction error and the simplicity of the model. Gamma is the other hyper-parameter of SVM regarding the Gaussian function inside the RBF kernel. Performance of SVM is very sensitive to changes in this parameter. Tested values for hyper-parameter C were 0.001, 0.1, 1, 5, and 10 and for parameter Gamma 0.005, 0.05, 0.5, and 5. The “e1071” R package was used for the analyses (Meyer et al., 2019).

Genomic BLUP (GBLUP) was used as a reference predictive method, and it was implemented to assess predictive performance within dPB dataset (trn.1-tst.1) and to assess its performance for predicting average CB offspring performances from PB sires genotypes among the other scenarios. In all cases, the same outer training and testing datasets partitions than those used with SVM were used. The GBLUP is a genome enabled the best linear unbiased prediction model (VanRaden, 2008). GBLUP uses genomic relationships to estimate the breeding values of the individuals. The genomic relationship matrix was computed with the 46,610 SNPs available (VanRaden, 2008) and included all animals involved in each scenario. Variance components in each scenario were estimated using Gibbs2f90 software (Misztal, 1999). Single chains of 250,000 iterations were run by discarding the first 25,000. Samples of the parameters of interest were saved every 10 iterations. Then, for each scenario, predicted phenotypes in the corresponding folds were the BLUP solutions obtained with Blupf90 software (Misztal, 1999) using the previously estimated variance components.

## Results

Predictive performance of all SVM reached a maximum within the range of SNP subset sizes investigated, suggesting that increasing the SNP subset size beyond 2,000 features would not increase the model prediction performance for the dataset structure and characteristics of this study.

### Prediction Performance of Individual Purebred Records

**Figure 2:**
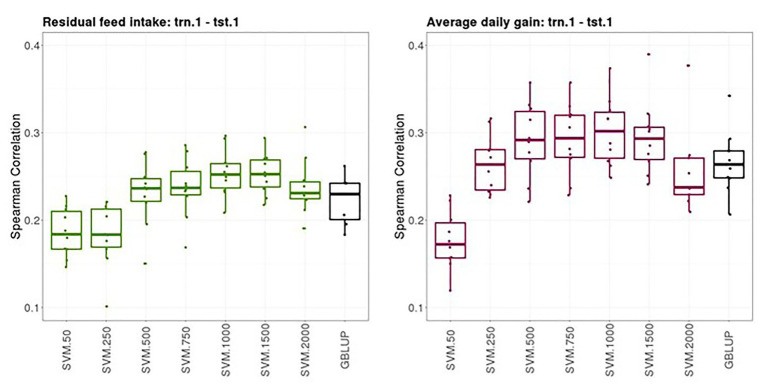
Boxplots of the Spearman correlations between observed and predicted residual feed intake and daily gain at fattening records obtained with genomic BLUP (GBLUP) and support vector machine (SVM) using different subset sizes of single nucleotide polymorphisms (SNPs) as predictor variables in a 10-fold cross-validation for scenario trn.1-tst.1 (see Figure 1 for a description).

Figure 2 shows boxplots of the Spearman correlations between observed and predicted RFI and ADG records obtained from a 10-fold cross-validation in trn.1-tst.1 scenario with GBLUP and SVM with different SNP subsets. The median SC (IQR, in square brackets) between predicted and yet-to be observed PB records across testing sets obtained with GBLUP was 0.23 [0.04] for RFI and 0.28 [0.03] for ADG. The highest predictive performance obtained with SVM was 0.25–0.26 [0.03] for RFI with a subset of 500, 750, or 1,000 SNPs and 0.30 [0.05] with a subset of 500 SNPs for ADG. In both traits, the prediction performance was slightly higher with SVM combined with an appropriate SNP subset than with the standard GBLUP that used all available SNPs after quality control.

### Prediction Performance of Average Crossbred Offspring Records

**Figure 3:**
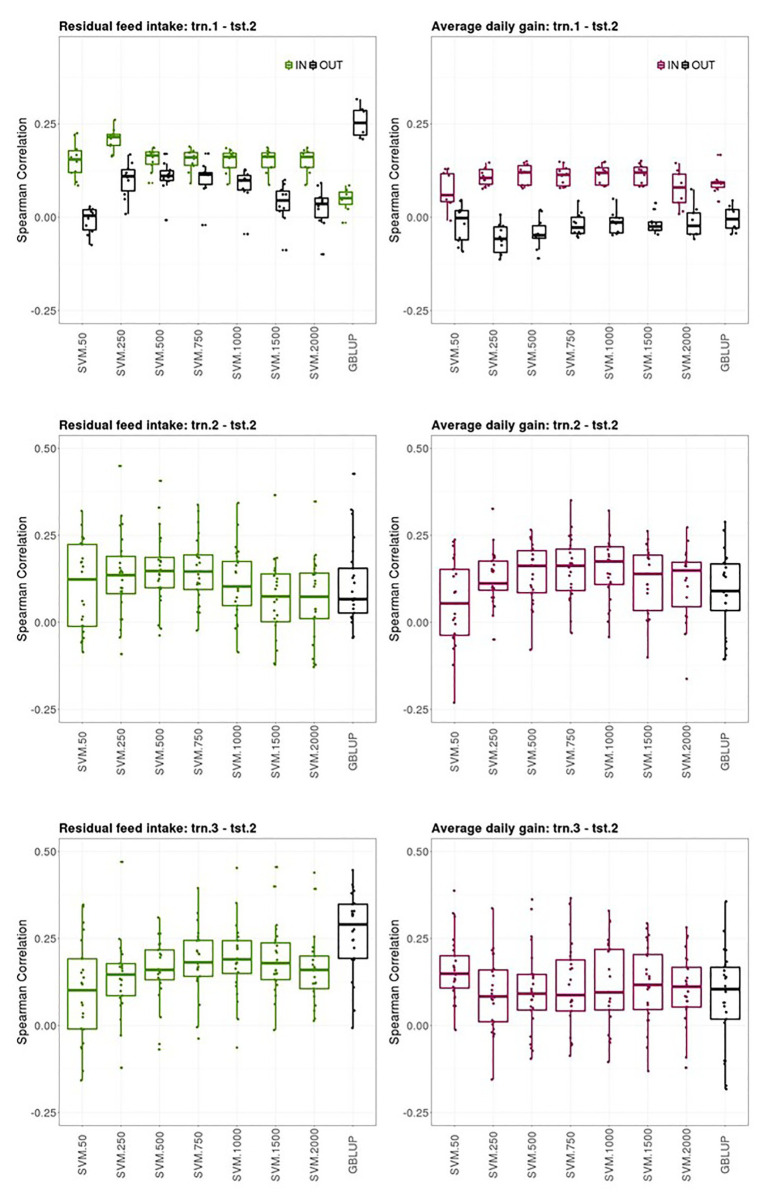
Boxplots of the Spearman correlations between observed and predicted residual feed intake and daily gain at fattening records obtained with GBLUP and SVM using different subset sizes of SNPs as predictor variables in a *k*-fold cross-validation for scenario trn.1-tst.2 (**upper panel**), trn.2-tst.2 (**middle panel**), and trn.1-tst.2 (**lower panel**). See Figure 1 for scenario description. In scenario trn1.tst2, “IN” refers to the situation in which the sires have their own performance in the training set and “OUT” refers to the opposite situation.

Prediction performances of several models fitted using different sources of information in the training set for predicting average CB offspring performances from PB sires genotypes are presented in this section. Figure 3 shows boxplots of the Spearman correlations between observed and predicted RFI and ADG records obtained in the tst-2 with SVM combined with increasing SNP subset sizes and GBLUP in the trn1.1-tst.2, trn.2-tst.2, and trn.3-tst.2 scenarios.

The ability of the PB sire genotype to predict their average CB offspring performance when the SVM model was fitted using individual PB phenotypes and genotypes (trn.1-tst.2 scenario, upper panels Figure 3) substantially differed between the sires that appeared themselves in the trn.1 (i.e., their individual performance is included in trn.1) from those who did not. The number of sires that contributed to the model fitting in trn.1 with their own PB performance was on average (SD) across the 10-fold 120.6 (3.8) out of 257 sires available. For RFI, the highest predictive ability of CB offspring records of the sires having their own performance in the training set was obtained with SVM and 250 SNPs (0.22 [0.03]) then, increasing the number of SNPs reduced the predictive performance. For ADG, the highest SC median was obtained with SVM and 500, 1,000, or 1,500 SNPs (0.12 [0.05]), and then with 2000 SNPs, SC was reduced. With SVM, the highest predictive ability of average CB offspring records of the sires that did not have their own performance in the training was obtained with 250, 500, or 750 features for RFI (0.11 [0.03–0.06]), whereas it was null for ADG. GBLUP showed also no predictive ability for ADG for the “OUT of training sires” and very poor prediction ability for the “IN training sires” (0.10 [0.02]). However, for RFI, GBLUP showed the highest predictive ability of all models for the “OUT of training sires” (0.25 [0.07]), whereas predictive ability for the “IN training sires” was low. On average, the stability of the results was better for sires having individual records in the training sets than for un-recorded sires across models.

The ability of the PB sire genotype to predict their average CB offspring performance improved when model was fitted using the same target trait and features used for the predictions (trn.2-tst.2 scenario, middle panels Figure 3). The highest predictive ability was obtained with 500 or 750 SNPs for RFI (0.15 [0.09]) and with 1,000 SNPs for ADG (0.17 [0.11]). Predictive ability of GBLUP was lower than the obtained with the best SVM model for both traits: 0.08 [0.12] for RFI and 0.09 [0.11] for ADG. The stability of the predictions was low in this scenario, given the large IQR obtained for the SC values across testing sets and models in both traits, which can lead to quite good or quite bad predictions (SC ranging from −0.12 to 0.45 for RFI and from −0.23 to 0.48 for ADG depending on the testing set).

Finally, the ability of PB sire genotypes to predict their average CB offspring performance from models fitted with individual CB information (trn.3-tst.2) is presented in Figure 3 (lower panels). The highest predictive performance for RFI was obtained with GBLUP (0.29 [0.16]) followed by SVM with 1,000 features (0.19 [0.09]), whereas the highest for ADG was obtained with SVM with only 50 features (0.15 [0.09]). Prediction ability with GBLUP was of 0.10 [0.15] for ADG. Like in trn.2-tst.2 scenario, the interquartile ranges of the SC across testing sets in the trn.3-tst.2 scenario were large, showing the instability of the prediction obtained using these datasets (SC ranging from −0.16 to 0.47 for RFI and from −0.15 to 0.39 for ADG depending on the testing set).

### Prediction Performance in the Youngest Generations

In this section, the prediction ability of the models used to predict average CB offspring performances from the youngest generations with the different scenarios trained on previous generations are presented.

**Figure 4:**
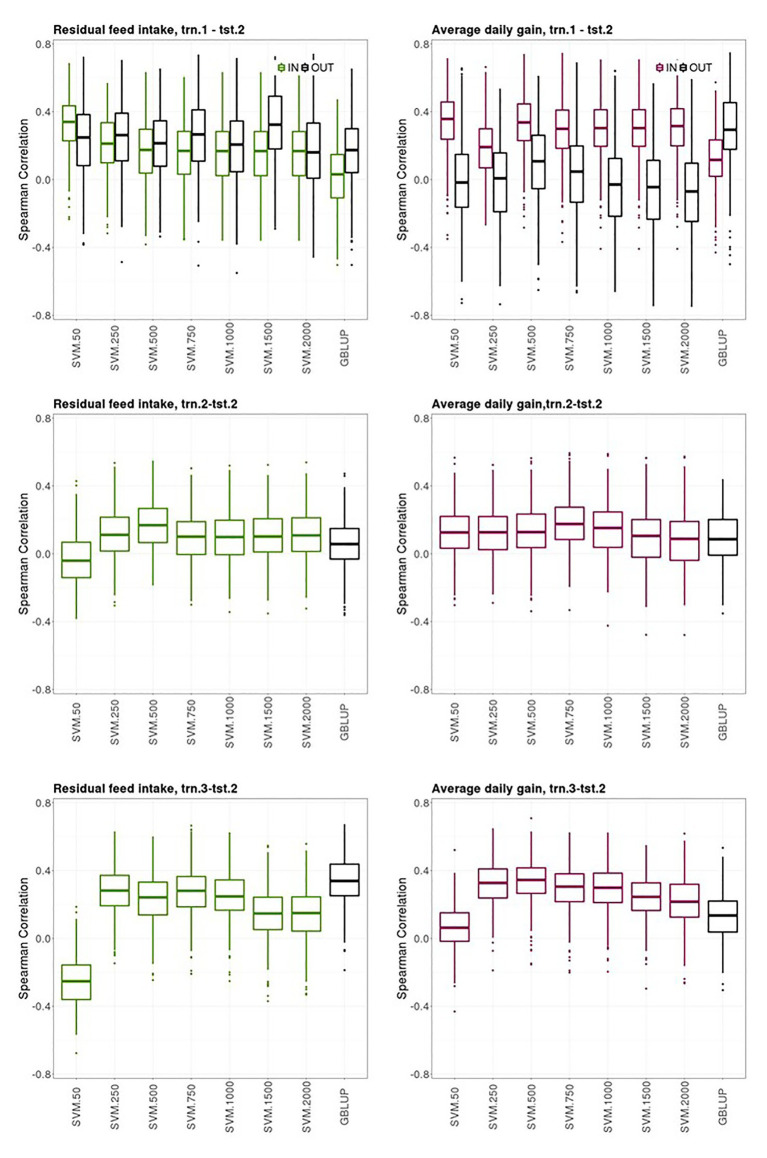
Box plots for the bootstrap distribution of Spearman correlations between observed and predicted residual feed intake and daily gain at fattening records obtained with Genomic BLUP (GBLUP) and support vector machine (SVM) using different subset sizes of SNPs as predictor variables in different scenarios. Testing sets were all composed of animals from the last two generations while training sets contained information from animals belonging to all previous ones. See Figure 1 for scenario description. In scenario trn1.tst2, “IN” refers to the situation in which the sires have their own performance in the training set and “OUT” refers to the opposite situation.

Figure 4 shows boxplots of the Spearman correlations between observed and predicted RFI and ADG records obtained with the bootstrap sampling in the testing sets of trn.1_OLD_-tst.2_YOUNG_, trn.2_OLD_-tst.2_YOUNG_, and trn.3_OLD_-tst.2_YOUNG_ scenarios with SVM using an increasing number of the most informative SNP as predictor variables and GBLUP. The presence of own individual PB phenotype and genotype in the training set improved the prediction ability of the PB sire genotype to predict its young CB offspring performance for ADG but not for RFI, where both groups of sires had similar prediction performances (“IN training sires” vs. “OUT of training sires” in trn.1_OLD_-tst.2_YOUNG_, Figure 4, upper panels). The highest median SC (IQR in brackets) between predicted and yet-to be observed average adjusted CB offspring records for the “IN training sires” obtained with SVM was 0.34 [0.21] and 0.36 [0.22] for RFI and ADG, respectively, with 50 SNPs. The highest median SC (IQR in square brackets) obtained for the “OUT of training sires” with SVM was of 0.33 [0.31] with 1,500 SNPs for RFI and 0.11 [0.31] with 500 SNPs for ADG. The median SC for the “OUT of training sires” obtained for GBLUP was 0.17 [0.26] and 0.30 [0.29] for RFI and ADG, respectively. The median SC for the “IN training sires” obtained for GBLUP was null for RFI and 0.12 [0.21] for ADG.

The ability of the youngest PB sires to predict their average CB offspring performance with their genotypes when model was fitted using the same target trait and features from previous generations (trn.2_OLD_-tst.2_YOUNG_ scenario, middle panels from Figure 4) was of 0.17 [0.20] and 0.18 [018] with SVM with 500 and 750 SNPs, for RFI and ADG, respectively. This scenario leads the poorest predictive CB offspring performance compared to the other two ones. GBLUP showed the same poor prediction ability: 0.06 [0.18] for RFI and 0.09 [0.22] for ADG.

The ability of the youngest PB sire genotypes to predict their average CB offspring performance from models fitted with individual CB information from previous generations (trn.3_OLD_-tst.2_YOUNG_, upper panels from Figure 4) was 0.28 [0.18] for RFI with SVM and 250 or 750 SNPs and 0.34 [0.15] for ADG with SVM with 500 SNPs. In this scenario, predictive performances were null with SVM combined with the smallest SNPs subset. Predictive performance of GBLUP was 0.34 [0.17] for RFI and 0.14 [0.18] for ADG.

Finally, a general trend was observed. The SVM models that showed the highest prediction ability and stability across the k-fold cross-validations in the three scenarios (i.e., prediction performance in tst.2 from model fitting in trn.1, trn.2., and trn3, Figure 3), also gave good predictions in the youngest generations (tst.2_YOUNG_) when models were fitted with data from older generations (trn.1 _OLD_, trn.2_OLD_, and trn3 _OLD_, Figure 4). However, the clearly higher prediction performance of CB offspring of the “IN training sires” compared to the “OUT of training sires” in the 10-fold-CV (trn.1-tst.2, Figure 3) was not clearly denoted when data was split according to OLD and YOUNG generations (trn.1_OLD_‐ tst.2_OLD_, Figure 4).

## Discussion

One of the major benefits of implementing GS in pig breeding is that elite boars in nucleus herds can be evaluated on traits recorded on animals that even do not bear any kinship with them. Traits related to CB performance, whose genetic improvement is crucial in pig crossbreeding schemes (Meuwissen et al., 2016), are among them. In this research, the use of different sources of information to predict CB performance to evaluate PB candidates for RFI and ADG with reduced SNP subsets was explored using SVM. Its prediction performance was compared to that of GBLUP, used as benchmark.

SVM models have been used in genome-wide prediction due to their ability to deal with potential non-linearity between features and target traits in animals and plants (Moser et al., 2009; Long et al., 2011; Montesinos-López et al., 2019). Our results indicate that SVM regression models were efficient in terms of prediction performance even when using a reduced subset of SNPs. This implies that low-density SNP panels could be cost-effective for breeding programs, since many animals could be genotyped at low cost, leading to a potential increase in selection intensity. In addition, feature selection (i.e., selection of a subset of predictor variables from the input data) reduces computation requirements and adverse effects on prediction performance of irrelevant variables due to over-fitting, which is especially an important problem in studies with high-dimensional/high-throughput data (Chandrashekar and Sahin, 2014). Feature selection was performed here in each outer training set using the rank correlation between the target trait and the SNP prediction. Selection of markers must be done using training set data only and must be repeated at each replication of the cross-validation when a new training dataset is encountered. If feature selection is done using the whole dataset before cross-validation, biased estimates of model accuracy are obtained (Hastie et al., 2009). In addition, when features have a high level of redundancy, different training samples can lead to different feature ranks (and, therefore, different subsets of features), which yield the same prediction accuracy. In order to design a low-density SNP panel for genetic selection or diagnostic, the stability of feature selection methods is important. The agreement of prediction models produced by an algorithm when trained on different training sets is known as “preferential stability” (Somol and Novovicova, 2010). Therefore, it is important to use a feature selection method that achieves a good prediction performance on independent data sets but that also produces a stable set of predictors, this understood as subsets that are less sensitive with respect to changes in the training set. The choice of method also depends on the available computational resources. It is desirable to evaluate feature selection methods for each specific problem/dataset because there is no group of methods that outperforms all other ones in every dataset (Somol and Novovicova, 2010; Haury et al., 2011; Bommert et al., 2020). In this study, rank correlation was chosen as metric based on his behavior when using data from scenario trn1.tst1.

### Prediction Performance of Individual Purebred Records

Within PB animals (trn.1-tst.1 scenario), SVM with an optimal number of selected SNPs outperformed the predictive performance of the benchmark model (GBLUP) in the two traits analyzed (Figure 2). Phenotype prediction using GBLUP is performed through the use of genomic breeding values obtained from the additive combination of all SNP marker effects simultaneously (Meuwissen et al., 2001). In our study, GBLUP using all SNPs available was the benchmark model. Further research could be to test predictive performance of GBLUP using subsets of the most informative SNPs. The GBLUP has been successful for selection purposes in many breeding programs (de los Campos et al., 2013; Meuwissen et al., 2016). However, its parametric assumptions are not always met and other more flexible approaches may attain better predictive accuracies (Gianola et al., 2006). The genetic basis of target phenotypes is a major factor affecting differences in prediction accuracy between parametric and non-parametric methods. For instance, SVM and other non-parametric models outperformed parametric models when epistasis influences phenotypes in a simulation study (Howard et al., 2014). This is because non-parametric models can deal with interactions among predictor variables and non-linear relationships with the target variable, (but without explicitly modeling these interactions or functional forms). Nevertheless, using such methods for selection purposes in a classical framework is not straightforward. This is because coefficient estimates are difficult to interpret, precluding quantification of additive genetic variance. However, if these methods provide a good prediction performance due to their ability to capture genetic effects in the broad sense (including additive genetics effects), their potential in GS cannot be ignored.

### Prediction Performance of Average Crossbred Offspring Records

In the scenarios used to test the ability to predict CB offspring performance from PB sire genotypes, results suggested that the best SVM models (in terms of prediction quality and stability of results) gave good predictions of average CB offspring records of young candidates using a model fitted with information from previous generations. However, predictive performance results in the “YOUNG/OLD scenarios” should be taken with caution because only a single realization was performed in each comparison. The bootstrap approach performed in the testing sets, provides only an approximate uncertainty measurement of prediction accuracy. Ideally, learners must be tested across several realizations of independent training/testing data sets.

Scenarios trn.1-tst.2 and trn.1_OLD_-tst.2_YOUNG_ assessed the ability to predict CB performance in a context, where only PB information is used to fit the model. This is classical in pig crossbreeding schemes, where genetic improvement of CB traits is expected to occur as a correlated response to genetic improvement in PB traits. The ability of the PB sire genotype to predict average CB offspring performance when the prediction model was fitted using individual PB phenotypes and genotypes was low for both RFI and ADG (trn.1-tst.2 scenario, Figure 3). In this scenario, the PB genotype was used to predict a different response/target trait in the training and in the testing datasets (individual phenotype vs. average CB progeny phenotype). Thus, predictions obtained in tst.2 somehow reflect that genetic differences within a PB line do not produce similar changes in the CB population, as estimated correlations between PB and CB traits suggested (Wientjes and Calus, 2017). However, prediction of CB offspring performance was systematically better for sires that had their own record in the training set (“IN training” sires), than for sires lacking records in the training set (“OUT of training” sires), where predictions were very poor. The best SVM model outperformed prediction ability of GBLUP except for predicting “OUT of training” sires CB performances were a quite high an unexpected predictive accuracy was found for RFI using GBLUP. Unfortunately, we cannot find a suitable explanation for the higher predictive performance for the “OUT of training” sires with respect to the “IN training sires” for RFI. We would expect that sires recorded in the training would get better predictions of their CB offspring in the testing set, as it has been the tendency for all the SVM models and for GBLUP in the other trait. A PCA biplot with the two first principal components of the G matrix did not reveal any hidden population structure involving trn1 and IN and OUT of training tst2 individuals, that could explain this result (not shown).

When evaluating the models under more realistic conditions of selection (trn.1_OLD_-trn.2_YOUNG_, Figure 4), predictions of CB performance of the “IN training sires” were improved for ADG, while remaining of similar magnitude for RFI. The very poor predictions achieved for the young “OUT of training” sires suggests that the strategy to evaluate PB lines for CB performance that leads to the shortest generation interval and reduced genotyping efforts is clearly far from being feasible for ADG. However, it could be an option for improving RFI, because moderate prediction performances were obtained either for the “IN training” or the “OUT of training” YOUNG sires. Nevertheless, when the own individual performance of the young PB sire was included in the data used to fit the model, (which reduces the response to selection per time units) an acceptable but low prediction quality would be attained in its yet-to-be observed CB offspring (“IN training sires” from trn.1_OLD_-tst.2_YOUNG_, Figure 4), specially for ADG. Therefore, candidates for selection can be evaluated for their yet-to-be observed CB offspring performance right after their own RFI and ADG performances are available. This is of interest for traits recorded in selection candidates that are usually evaluated at the end of the fattening period (at about 160 days of age, Tribout et al., 2013), such as RFI and ADG. This implies that the evaluation for CB performance would not require maintaining costs of the candidates until they would have progeny CB records and candidates could be evaluated for both PB and CB performance simultaneously, which makes possible to include CB traits in selection decisions of the PB lines. However, it is important to note that the accuracy of prediction obtained for the “IN training sires” is probably the result of genetic relationships captured by the marker instead of improved accuracy due to linkage disequilibrium between the genes and the markers, as shown by Habier et al. (2007). This could explain the low prediction accuracy obtained when individuals in the testing set were not directly related with individuals in the training set. Habier et al. (2007) recommended consideration of the accuracy of predictions from several generations after marker estimation, and not only from a single generation if the objective is to make predictions over some generations after estimation of marker effects. No substantial differences in prediction ability were encountered between GBLUP and SVM models for most of the cases evaluated in trn.1_OLD_-tst.2_YOUNG_. However, GBLUP gave better prediction ability for predicting ADG offspring records of “OUT of training” young PB sires than the best SVM model.

In the presence of genotype by environment and genotype × genotype interactions, PB performance can be a poor predictor of CB offspring performance, so the use of a CB population as training dataset is advisable (Dekkers, 2007; Zeng et al., 2013; Esfandyari et al., 2015, 2016). Thus, another strategy would be to have a reference population including genotyped and phenotyped CB individuals to fit the model and then to evaluate PB candidates for their yet-to-be observed CB offspring performance using their own genotypes. This approach was assessed in trn.3-tst.2 scenario, which leads to a prediction quality similar to the best situation in trn1.tst2. Prediction of average CB offspring performance of the youngest PB sire using their own genotypes with the best models fitted with individual CB information from previous generations (trn.3_OLD_-tst.2_YOUNG_) was good and close in magnitude to that obtained for the “IN training sires” using PB data for training (trn.1_OLD_-tst.2_YOUNG_), specially using GBLUP. This means that with a CB reference population for model fitting, PB candidates can be evaluated for CB performance at an early age, right after being genotyped. This strategy would reduce generation interval, but at the cost of also genotyping CB individuals. Alternatively, the reference population could be composed of a mixture of PB and CB animals, in order to get a more representative collection of genetic effects and interactions. Other strategy could be implementing a multi-label prediction model jointly considering PB and CB information. Exploring such strategies is a subject for further research. In a simulation study, Esfandyari et al. (2016) concluded that training a parametric GS model accounting for dominance effects using CB data led to greater phenotypic response at the CB level compared to training the model on PB lines.

The idea behind scenario trn.2-tst.2 was to fit a prediction model using the same genotype and phenotype information than what was intended to be predicted on PB candidates, assuming that a phenotype expressed in PB animals was not necessarily under the same gene action as a phenotype expressed in CB animals. This scenario requires progeny records from the PB sires available in the training dataset, lengthening the generation interval. The resulting prediction ability with SVM models, although still low, was slightly better than the one obtained with the models fitted with trn.1 (trn.2-tst.2 vs. trn.1-tst.2, Figure 3). This could be due to the fact that genes or effects involved in growth rate and feed efficiency differ between PB and CB populations, in which using same information for model fitting and prediction is advisable. The instability encountered in the predictions of trn2.tst.2 scenario can be explained by the small amount of data available in this dataset (i.e., only 257 average CB records were available). This problem was not compensated by the better predictive performance expected for an average than for a single data point. It would be expected that averaging CB offspring records per sire would average out dam effects and other environmental effects not accounted for in the data pre-adjustment. Prediction accuracy of the youngest PB sire generations was very poor with all models, possibly because a low level of relatedness between individuals of the training and testing sets (Habier et al., 2007). SVM models outperformed the prediction performance of GBLUP.

Our research was mostly focused in finding a prediction model suitable for improving a terminal sire line for growing-finisher pigs CB performance, with effects from maternal lines (i.e., effects of CB dams) ignored. To our knowledge, this is the first evaluation made of a non-parametric approach for predicting CB phenotypes from SNP genotypes. In a two‐ or three-way crosses context, the advantage of using a non-parametric over a parametric approach is that the first does not need to explicitly specify non-additive genetic effects (such as dominance and epistasis) nor to account for potential non-linear relationships between genotypes and phenotypes. We could show that the tested models could outperform the benchmark GBLUP in some of the scenarios explored, opening promising future axes of research to refine the use of these methodologies in crossbreeding genomic evaluations.

## Conclusion

SVM is an efficient method for predicting average RFI and ADG CB performances from PB sire genotypes using a selected subset of SNPs (250–1,000). This makes SVM appealing for select candidates to selection of PB sire lines for improved CB performance with low-density SNP chip panels. Given the predictive performance of SVM in the scenarios explored, selection candidates could be evaluated for CB performance after collection of their own RFI and ADG performances in a classical pig crossbreeding scheme framework or sooner right after being genotyped using a reference population of CB animals. Genetic progress and economic impact of these approaches need to be addressed.

## Data Availability Statement

The data analyzed in this study is subject to the following licenses/restrictions: the datasets presented in this article are not readily available because of containing information that could be used by commercial competitors. Topigs Norsvin (Beuningen, Netherlands) fully agrees with transparency in science and even welcome alternative analysis approaches on the data with a gatekeeper, a trusted person, who considers the relevance and the motives of the people interested in the data. Requests to access these datasets should be directed to Rob Bergsma, Rob.Bergsma@topigsnorsvin.com.

## Ethics Statement

Ethical review and approval was not required for the animal study because all data used in this study were obtained from existing database made available by Topigs Norsvin (Beuningen, Netherlands). Therefore, no Animal Care Committee approval was necessary for the purposes of this study.

## Author Contributions

LT and MP conceived and designed the study, carried out the analyses, and wrote the original draft. RB prepared and provided the raw data. DG and HG provided critical insights and gave methodological suggestions. All authors discussed the results, reviewed, and approved the final manuscript.

### Conflict of Interest

RB was employed by the company Topigs Norsvin (Beuningen, Netherlands).

The remaining authors declare that the research was conducted in the absence of any commercial or financial relationships that could be construed as a potential conflict of interest.
